# Dietary zinc requirement of juvenile stinging catfish *Heteropneustes fossilis* based on growth performance, haematology, and tissue mineral composition

**DOI:** 10.1016/j.heliyon.2024.e28422

**Published:** 2024-03-20

**Authors:** Muslima Akter Lima, Md. Amzad Hossain, Md. Rabiul Islam, Md. Nur Amin Mukul, Taslima Akter

**Affiliations:** Department of Aquaculture, Bangabandhu Sheikh Mujibur Rahman Agricultural University, Gazipur, 1706, Bangladesh

**Keywords:** Zinc requirement, *Heteropneustes fossilis*, Growth performance, Feed conversion ratio, Haematology, Bone mineralization

## Abstract

This investigation was done to determine how much zinc (Zn) the stinging catfish, *Heteropneustes fossilis*, needs in its diet. Five isonitrogenous (34.5% protein) and isolipidic (6.0% lipid) diets were prepared to contain graded levels of Zn (0, 10, 20, 30, and 40 mg kg^−1^), supplied as zinc sulfate (ZnSO_4_·7H_2_O), and referred to as Zn0, Zn10, Zn20, Zn30, and Zn40, respectively. A total of 600 fish (initial body weight: 1.41 ± 0.02 g) were stocked in 15 glass aquaria (40 fish/aquarium), each with 180 L water capacity. For ten weeks, each diet was hand fed to three groups of fish twice daily until they appeared satisfied. The highest weight gain and specific growth rate, and lowest feed conversion ratio were recorded in fish fed with a 30 mg Zn kg^−1^ diet. Zn contents in bone and muscle linearly increased up to 30 mg kg^−1^ Zn and then remained stable, while iron (Fe) and copper (Cu) contents in bone and muscle had an inverse pattern with the inclusion level of dietary Zn. Increasing dietary Zn levels up to 30 mg kg^−1^ was found to improve values of hematological parameters such as red blood cell (RBC), white blood cell (WBC), haemoglobin (Hb), and haematocrit (HCT). These values, however, decreased when the dietary Zn level was further increased. The serum alkaline phosphatase level was the highest in fish fed a diet containing 30 mg kg-1 of Zn. Regression analyses based on weight gain, specific growth rate, and bone and muscle Zn concentrations indicated that the optimum dietary Zn requirement for stinging catfish was in a range of 27.4–36.5 mg kg^−1^.

## Highlights

• Best growth performance and feed conversion ratio was recorded at 30 mg Zn kg^−1^ diet.
• Zn contents in bone and muscle linearly increased to 30 mg kg^−1^ Zn and then stabilized.
• Serum alkaline phosphatase level peaked at 30 mg kg^−1^ of dietary Zn.
• A 30 mg Zn kg^−1^ diet was most recommendable for stinging catfish.
• However, based on regression analysis optimum dietary Zn requirement was 27.4–36.5 mg kg^−1^.

## 1 Introduction

The stinging catfish (*Heteropneustes fossilis*), an omnivorous, freshwater catfish, appears to have considerable potential as a farmed fish because it survives and grows well in captivity at high stocking densities, and in water that would compromise the farming of many other species [1]. It has a favourable nutritional profile regarding muscle proteins, lipids, and minerals and is readily accepted by consumers [2,3]. These characteristics have stimulated interest in farming the stinging catfish on the Indian subcontinent, and production volumes are increasing [4]. However, lacking a balanced feed is the major challenge in stinging catfish farming [5].

In many aquaculture farms, feed accounts for more than 70% of the variable operational costs [6]. Therefore, it is crucial to have a solid understanding of nutrition and specific nutritional requirements of fish for successful aquaculture. The fish will not grow well if there is a nutritional shortage in the diet. Thus, the production of fish requires nutritionally balanced diets of the highest possible quality [7,8]. Understanding fish's various nutritional needs is crucial to formulate a quality diet. Among various nutrients, the mineral is one of the vital nutrients that must be considered. Mineral requirements in fish diets are typically extremely low; however, they are essential for the nutrition of fish [9,10]. Although fish are capable of absorbing most of the minerals from surrounding water, the primary source is diet [7,11]. Despite the growing awareness of the critical role of minerals in a variety of life processes, including skeletal development, organ function, and healthy growth, research on the mineral requirement of fish has advanced slowly [12]. According to Lall and Kaushik [11] zinc (Zn) is regarded as one of the most crucial trace elements among the essential minerals needed by fish for growth, metabolism, bone development, gene regulation, and energy production. Due to its involvement in many different biochemical pathways, it is the only metal found in all six enzyme types and the second-most prevalent transition metal in organisms [13]. It controls numerous crucial physiological processes, such as the metabolism of proteins, lipids, and carbohydrates [14]. It functions as a cofactor in several metalloenzymes, including carboxypeptidases, alcohol dehydrogenases, carbonic anhydrases, glutamate dehydrogenases, superoxide dismutases, and DNA and RNA polymerases [[15], [16], [17]]. Therefore, dietary Zn deficiency impairs hampers the physiological and biochemical pathways in the fish body, leading to poor growth.

Dietary Zn deficiency may cause cataracts, skin issues, decreased growth, appetite loss, low zinc levels in the bone and serum, and dwarfism in fish [11,18]. Dietary Zn level could also affect fish's survivability and whole-body mineral content [15]. During the digestive process when Zn is absorbed, Zn competes with other bivalent minerals, including cadmium, iron, copper, and calcium, for binding sites and can be harmful to fish when taken in excess [17,19,20]. Elevated Zn levels could trigger branchial mucus secretion that affects ion regulation and other essential gill-based functions like oxygen (O_2_) uptake. Aside from the effects on respiratory function and ion regulation, dietary Zn may also influence oxidative stress markers [21]. Buentello et al. [22] stated that unnecessarily high supplementation of Zn in diet should be limited to reduce the minerals load in the aquatic environment. As a result, fish require an optimum amount of dietary Zn supplementation to maintain excellent growth performance and good health.

So far, the Zn requirements of several fish species have been documented; however, requirements differ considerably depending on the species of fish and life stage [12,15,19,[23], [24], [25]]. Although the requirements of some minerals such as manganese [26], iron [27], and phosphorus [5] have been reported for stinging catfish; however, to our knowledge, no information is available on Zn requirement. Therefore, this study aimed to assess growth performances, haematological parameters, muscle and bone mineralization, and serum alkaline phosphatase activity to establish the optimum dietary Zn requirement of stinging catfish.

## 2 Material and methods

### 2.1 Preparation of experimental diet

Five isonitrogenous (34.5% protein) and isolipidic (6.0% lipid) diets were formulated from purified ingredients. Table 1 lists the detailed formulation and proximate composition of the experimental diets. Vitamin-free casein (38%) was used as a dietary protein source. Corn oil (7%) and fish oil (5%) were the primary dietary lipid sources. Corn starch (38%) and α-Cellulose (4%) were used as the carbohydrate source. Zinc sulfate (ZnSO_4_·7H_2_O, Sigma Chemical) were included at the expense of α-cellulose to obtain diets with graded levels of Zn (0, 10, 20, 30, and 40 mg kg^−1^) and referred to as Zn0 (Control), Zn10, Zn20, Zn30, and Zn40, respectively. After that, a food mixer was used to mix all the feed ingredients properly. While mixing the ingredients, the pre-blended fish oil and corn oil premixes were added gradually. Double-distilled water was used to dissolve the zinc sulfate (ZnSO_4_·7H_2_O) for each diet before mixing it with the other ingredients to form a thick dough. The dough was subsequently processed through a pelletizer with a 2 mm diameter die and air-dried to approximately 10% moisture at room temperature. Finally, the experimental diets were packed in plastic bags, sealed, and stored at −20 °C until further use.

**Table 1:** Formulation and proximate composition (on dry weight basis) of the experimental diets.

Ingredients (%)	Treatments				
	Zn0	Zn10	Zn20	Zn30	Zn40
Vitamin free casein^a^	38.00	38.00	38.00	38.00	38.00
Corn starch	38.00	38.00	38.00	38.00	38.00
Corn oil	7.00	7.00	7.00	7.00	7.00
Fish oil	5.00	5.00	5.00	5.00	5.00
Carboxymethylcellulose	3.00	3.00	3.00	3.00	3.00
Vitamin mix.^b^	2.00	2.00	2.00	2.00	2.00
Zinc-free mineral mix.^c^	3.00	3.00	3.00	3.00	3.00
Zinc-sulfate (ZnSO_4_·7H_2_O)	0.0000	0.00443	0.00885	0.01328	0.01771
α-Cellulose	4.0000	3.99557	3.99115	3.98672	3.98229
*Proximate composition (%)*					
Protein	34.36	34.58	34.51	34.79	34.67
Lipid	5.91	5.84	6.01	5.85	5.87
Ash	14.29	14.72	14.27	14.21	13.91
Moisture	11.53	11.27	11.31	11.57	11.31
Fiber	6.27	6.21	6.18	6.27	6.14

a Vitamin free casein (89% crude protein).

b Vitamin mixture supplied the following (mg/g mixture): thiamin hydrochloride, 5 mg; riboflavin, 5 mg; calcium pantothenate, 10 mg; nicotinic acid, 6.05 mg; biotin, 0.003 mg; pyridoxine hydrochloride, 0.825 mg; inositol, 10 mg; folic acid, 0.041 mg; L-ascorby1-2-monophosphate-Mg, 2.025 mg; choline chloride, 44 mg; menadione, 4 mg; alphatocopherol acetate, 3.35 mg; *para*-aminobenzoic acid, 5 mg; myo-inositol, 20 mg; retinyl acetate, 0.4 mg; cholecaliferol, 0.0004685 mg. All ingredients were diluted with alpha-cellulose to 1 g [29].

c Zinc-free mineral mixture supplied the following (mg/g mixture): FeSO_4_·6H_2_O, 2.125 mg; MgSO_4_, 137 mg; KCl, 75 mg; NaH_2_PO_4_, 87.2 mg; NaCl, 43.5 mg; AlCl3·6H_2_O, 0.15 mg; KI, 0.15 mg; CuCl_2_·2H_2_O, 0.1 mg; MnSO4·H_2_O, 0.80 mg; CoCl_2_·6H_2_O, 1 mg. All ingredients were diluted with alpha-cellulose to 1 g [29].

### 2.2 Experimental fish rearing methods and feeding management

Before the feeding study, fingerlings of stinging catfish were purchased from a commercial hatchery and acclimated to the rearing conditions for two weeks. During acclimatization, all the fish were kept in a large tank (600 L) with continuous aeration and fed on the experimental control diet without a Zn supplementation. Following the acclimatization period, fish with average body weight 1.41 ± 0.02 g were individually weighed, chosen, and distributed to the aquaria (40 ind./aquarium) to ensure that the overall biomass in each aquarium was comparable. The Zn concentration in the water fluctuated <0.02 mg L^−1^ without a significant variation between the aquaria over the trial period (*P* < 0.05). A total of 4 shelters for fish were set at the bottom of each aquarium. Each of the five experimental diets was fed to triplicate groups of fish to apparent satiation two times a day (9.00 and 16.00 h), 7 days a week for 10 weeks. Fish ingested the respective diet in less than 2 min; therefore, there was minimal Zn leaching into the rearing water. During the experiment, natural photoperiod (10L: 14D) was maintained. Each aquarium was supplied with aeration, and 30% of the water was replaced daily. Water quality parameters were monitored daily at 9:00 a.m. throughout the study period. The average water temperature was 27.75 ± 0.32 °C, dissolved oxygen concentration was 6.09 ± 0.2 mg L^−1^, pH value was 7.19 ± 0.13, and ammonia concentration was 0.12 ± 0.02 mg L^−1^.

### 2.3 Sampling

The fish underwent a 10-week feeding trial and were then starved for 24 h and given a dosage of tricaine methanesulfonate (MS-222) at 100 ppm as an anesthetic. All the fish were removed from the aquarium and counted using a scoop net. The body weight of each fish was determined by a digital electric balance (model EK600i). Blood samples were collected from 10 randomly selected fishes from each aquarium. Briefly, blood samples were taken from the caudal vein using a 1 ml tuberculin syringe dipped into a 2% heparin solution as an anticoagulant. Samples were then transferred to an EDTA (Ethylene Diamine Tetra Acetic Acid) tube (BD Microtainer®, UK) and kept for hematological analysis. For serum separation, an aliquot blood was collected in a serum separator tube (SST) and kept in room temperature for 30 min. The tube was centrifuged at 1500×*g* for 10 min and supernatant was collected for the determination of alkaline phosphate activity. Livers were collected from 10 fish after blood sampling. The adherent water of the liver was removed with the help of blotting paper, and then the weight was recorded. For mineral analysis, the muscle and bones were collected from the same fish used for blood and liver sample collection.

### 2.4 Determination of growth parameters and feed conversion ratio

The metrics below for feed conversion ratio and growth performance were determined at the end of the trial period following the formula:

Weight gain (g) = mean final weight (g) – mean initial weight (g)

$$\text{Weight}\;\text{gain}\;\left(\text{WG},\%\right)=\frac{\text{mean}\;\text{final}\;\text{fish}\;\text{weight}\;\left(g\right)–\;\text{mean}\;\text{initial}\;\text{fish}\;\text{weight}\left(g\right)}{\text{mean}\;\text{initial}\;\text{fish}\;\text{weight}\;\left(g\right)}\times 100$$

$$\text{Specific}\;\text{growth}\;\text{rate}\;\left(\text{SGR},\%\;\text{per}\;\text{day}\right)=\frac{\ln\;W2-\ln\;W1}{T2-T1}\times 100$$

where.

W_1_ = The initial live body weight (g) at time T_1_ (day)
W_2_ = The final live body weight (g) at time T_2_ (day)

$$\text{Feed}\;\text{conversion}\;\text{ratio}\;\left(\text{FCR}\right)=\frac{\text{Total}\;\text{feed}\;\text{consumption}\;\left(g\right)}{\text{Total}\;\text{body}\;\text{weight}\;\text{gains}\;\text{of}\;\text{fish}\;\left(g\right)}\times 100$$

$$\text{Survival}\;\text{rate}\;\left(\%\right)=\frac{\text{Final}\;\text{number}\;\text{of}\;\text{fish}\;\text{survived}}{\text{Number}\;\text{of}\;\text{actual}\;\text{fish}\;\text{stocked}}\times 100$$

$$\text{Hepatosomatic}\;\text{index}\;\left(\text{HSI}\right)=\frac{\text{Weight}\;\text{of}\;\text{liver}\;\left(g\right)}{\text{Weight}\;\text{of}\;\text{the}\;\text{body}\;\left(g\right)}\times 100$$

### 2.5 Proximate composition analysis

The proximate composition of the experimental diets was analyzed according to the Association of Official Analytical Chemists’ standard procedures [28]. Crude protein content was evaluated by auto Kjeldahl system (Model UDK159, VELP, Italy), and crude lipid content by ether-extraction method using a Soxhlet Extractor (Model 6XL, Soxhlet Extraction Apparatus, Bakhshi Co., Tehran, Iran). Moisture content was evaluated by the oven (Model FD 23, Binder, Germany) drying at 105 °C for 24 h, and ash content by using a muffle furnace (Carbolite RHF 17/6S, Carbolite Ltd., England) at 550 °C for 4 h.

### 2.6 Mineral analysis

Muscle and bone samples were collected from 10 fish to assess the selected mineral contents (Zn, Fe, and Cu) of stinging catfish*.* The fish were microwaved for 5 min, and the muscles from the bones (vertebrae and spines) were separated. After being cleaned with distilled-deionized water, the bones were dried at 105 °C for 2 h. The samples (muscle and bone) were digested in perchloric acid, and mineral analysis was performed utilizing techniques identical to Ref. [15] using an inductively coupled plasma mass emission spectrophotometer (ICP-MS, Thermo Fisher, USA). The operating conditions of ICP-MS were optimized for respective mineral.

### 2.7 Serum alkaline phosphatase analysis

An automatic biochemical analyzer (Sysmex-800, Sysmex Corporation, Kobe, Japan) and commercial diagnostic reagent kits (Sysmex Wuxi Co., Lt., Wuxi, China) were used to measure the activity of serum alkaline phosphatase.

### 2.8 Hematological analysis

A completely automatic haematology analyzer (Model: DH36, Dymind Biotechnology, China) was used to examine the blood samples in the Fish Nutrition Lab, Department of Aquaculture, BSMRAU to determine the haematological parameters such as leukocyte (WBC), erythrocyte (RBC), hematocrit (HCT), hemoglobin (Hb), mean corpuscular volume (MCV), mean cell haemoglobin (MCH), and mean corpuscular hemoglobin concentration (MCHC) by following the method used by Al-Mamun et al. [30].

### 2.9 Statistical analysis

The data were tested for normality and homogeneity of variances using the Shapiro-Wilk and Levene tests, respectively. The data were analyzed statistically by one-way ANOVA using the statistical software Statistix 10 (2013), and significance was indicated by the Least Significant Difference (LSD) option of the package, which was used to compare the means. The significance level was determined at *P* < 0.05. Furthermore, polynomial regression analysis was applied between weight gain (WG), specific growth rate (SGR), bone, and muscle Zn concentration (dependent variables), and different levels of dietary supplementation of Zn (independent variable) in the diet of stinging catfish.

## 3 Results

### 3.1 Growth performance, feed utilization, and survival

**Fig. 1:**
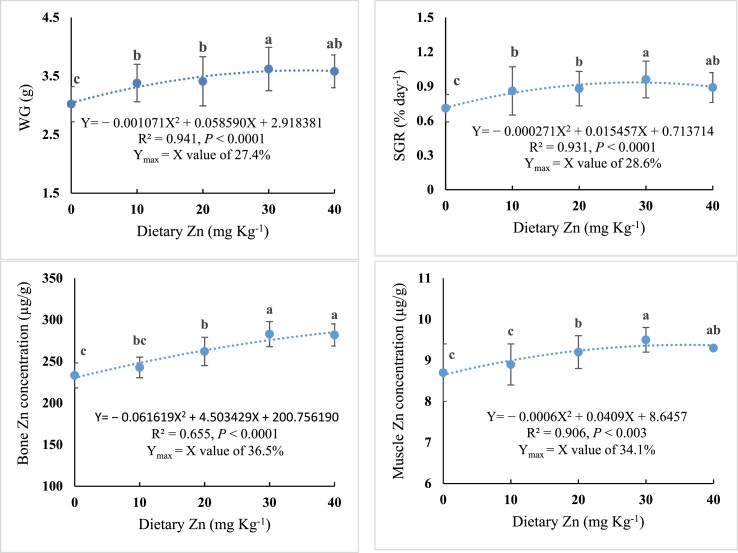
Polynomial regression analysis between weight gain (WG), specific growth rate (SGR), bone, and muscle Zn concentration (dependent variables), and different levels of dietary supplementation of Zn (independent variable) in the diet of stinging catfish.

Table 2 shows the growth performance and feed consumption data of stinging catfish following a feeding trial for 10 weeks. Weight gain (%) and specific growth rate (SGR % day^−1^) values increased following the enhancement of Zn level in experimental diets up to 30 mg Zn/kg diet; however, they did not increase with further enhancement in dietary Zn level. In regression analysis, a quadratic model was found to be the best-fit model between the inclusion level of Zn in the stinging catfish diet with WG (%) (Y = − 0.001071X^2^ + 0.058590X + 2.918381, *P* < 0.019 R^2^ = 0.941, Y_max_ = X value of 27.4%) and SGR (Y = − 0.000271X^2^ + 0.015457X + 0.713714, *P* < 0.0001, R^2^ = 0.931, Y_max_ = X value of 28.6%), respectively (Fig. 1). The results indicated that stinging catfish required 27.4–28.6 mg Zn kg^−1^ diet for optimum growth. On the other hand, FCR significantly (*P* < 0.05) decreased with increasing dietary Zn supplementation, reaching a minimum of 1.35 ± 0.18 in fish fed the diet containing 30 mg Zn kg^−1^ and then increased with further supplementation of Zn. HSI tended to increase as dietary Zn increased, and the highest HSI was observed in the fish fed diet containing 30 mg Zn kg^−1^. Though growth performance and FCR significantly vary with Zn supplementation level, no significant (*P* > 0.05) difference was observed in the survival rate of stinging catfish concerning different levels of Zn in the diet.

**Table 2:** Growth performance, feed conversion ratio and survival of stinging catfish fed with graded level of Zn for 10 weeks (Means in triplicate ± SD).

Index	Treatments				
	Zn0	Zn10	Zn20	Zn30	Zn40
IW (g)	1.41 ± 0.02	1.43 ± 0.01	1.41 ± 0.02	1.41 ± 0.01	1.42 ± 0.01
FW (g)	4.43 ± 0.30^c^	4.78 ± 0.32^b^	4.81 ± 0.42^b^	5.02 ± 0.37^a^	4.98 ± 0.28^ab^
WG (%)	214.18 ± 6.3^c^	234.27 ± 3.4^b^	241.13 ± 1.42^b^	256.03 ± 5.7^a^	250.70 ± 2.8^ab^
SGR (% day^−1^)	0.71 ± 0.12^c^	0.86 ± 0.21^b^	0.88 ± 0.15^b^	0.96 ± 0.16^a^	0.89 ± 0.13^ab^
FCR	1.59 ± 0.30^a^	1.42 ± 0.28^b^	1.38 ± 0.22^c^	1.35 ± 0.18^c^	1.45 ± 0.21^b^
HSI	1.26 ± 0.04^c^	1.41 ± 0.02^b^	1.42 ± 0.03^b^	1.58 ± 0.02^a^	1.42 ± 0.02^b^
Survival rate (%)	98.00 ± 0.54	98.00 ± 0.54	98.00 ± 0.54	100.00	100.00

* Data in the same row bearing different superscript letters indicate significant difference (*P* < 0.05). IW, initial weight (g); FW, final weight (g); WG (%), weight gain percentage; SGR, specific growth rate (% day^−1^); FCR, feed conversion ratio; HSI, hepatosomatic index.

### 3.2 Mineral concentration in tissues

Table 3 displays the mineral contents of bone and muscle tissues. Bone and muscle Zn concentrations of stinging catfish were significantly (*P* < 0.05) increased with the inclusion level of dietary Zn up to 30 mg Zn kg^−1^ and remained stable beyond this level. Fe and Cu contents in bone and muscle were also significantly altered by dietary Zn level (*P* < 0.05). The Fe and Cu content in bone and muscle decreased with the increasing supplementation of Zn in the diet. The relationship between bone and muscle Zn concentrations and dietary Zn level was expressed by a regression model, which indicated that the optimal dietary Zn level for the best bone and muscle Zn contents of stinging catfish was 36.5 and 34.1 mg Zn kg^−1^ diet, respectively (Fig. 1).

**Table 3:** Zn, Fe and Cu concentration in bone and muscle of stinging catfish fed with graded levels of Zn for 10 weeks (Means in triplicate ± SD).

Index	Treatments				
	Zn0	Zn10	Zn20	Zn30	Zn40
*Zn concentration (μg g*^*−*^*^1^)*					
Bone	233.2 ± 15.0^c^	242.8 ± 12.3^bc^	262.0 ± 17.0^b^	282.8 ± 15.1^a^	281.8 ± 13.3^a^
Muscle	8.7 ± 0.7^c^	8.9 ± 0.5^c^	9.2 ± 0.4^b^	9.5 ± 0.3^a^	9.3 ± 0.5^ab^
*Fe concentration (μg g*^*−*^*^1^)*					
Bone	234.2 ± 10.1^a^	232.6 ± 9.0^ab^	231.9 ± 8.6^b^	223.4 ± 8.2^c^	227.1 ± 8.8^c^
Muscle	135.2 ± 1.1^a^	133.2 ± 0.9^b^	132.3 ± 0.4^b^	130.3 ± 0.3^c^	126.9 ± 0.6^d^
*Cu concentration (μg g*^*−*^*^1^)*					
Bone	155.2 ± 5.1^a^	146.4 ± 2.4^b^	144.0 ± 4.8^bc^	142.8 ± 4.8^c^	145.8 ± 5.3^b^
Muscle	212.8 ± 8.8^a^	194.6 ± 8.0^b^	194.0 ± 8.6^b^	175.2 ± 5.8^c^	135.8 ± 5.3^d^

* Different superscript letters indicate significant difference (*P* < 0.05).

### 3.3 Hematological parameters

Table 4 represents the haematological parameters of stinging catfish at the end of 10 weeks feeding trial. Compared to the control diet (Zn0), the Zn-incorporated diets produced better haematological characteristics viz., Hb, HCT, and MCHC values were positively correlated (*P* < 0.05) with increasing levels of Zn up to 30 mg kg^−1^ diet (Zn30) and then levelled off. The RBC value gradually increased with the level of Zn supplement up to 30 mg kg^−1^ diet and then decreased. The maximum WBC value in this study was also obtained in the Zn30 treatment; however, further addition of Zn to the experimental diet showed decreasing trend. The lowest WBC and RBC values were recorded in the control (Zn0) group compared to all other treatments. Other haematological parameters, including MCV and MCH, showed decreasing features with the increasing levels of Zn in the diets.

**Table 4:** Hematological parameters of stinging catfish fed with graded levels of Zn for 10 weeks (Means in triplicate ± SD)^a^.

Index	Treatments				
	Zn0	Zn10	Zn20	Zn30	Zn40
RBC	1.32 ± 0.15^d^	1.42 ± 0.24^d^	2.04 ± 0.12^b^	2.55 ± 0.30^a^	1.83 ± 0.05^c^
WBC	27.92 ± 1.93^b^	29.21 ± 0.41^ab^	31.45 ± 1.34^ab^	33.26 ± 1.41^a^	30.81 ± 1.55^ab^
Hb	9.10 ± 0.18^b^	9.15 ± 0.16^b^	9.50 ± 0.23^b^	10.30 ± 0.25^a^	10.29 ± 0.18^a^
HCT	14.32 ± 0.98^b^	14.38 ± 0.42^b^	15.00 ± 1.09^ab^	15.64 ± 0.89^a^	15.35 ± 1.35^ab^
MCV	106.17 ± 0.69^a^	101.32 ± 1.03^ab^	73.51 ± 1.23^b^	61.13 ± 0.80^c^	83.67 ± 0.56^ab^
MCH	68.91 ± 1.69^a^	64.45 ± 1.23^b^	46.57 ± 1.56^d^	40.39 ± 1.31^e^	56.23 ± 1.23^c^
MCHC	63.350 ± 2.26^b^	63.63 ± 1.56^b^	63.63 ± 0.77^b^	65.85 ± 0.73^a^	67.04 ± 1.97^a^

a Data in the same row bearing different superscript letters indicate significant difference (*P* < 0.05). RBC: red blood cell ( × 10^6^ cells μL^−1^); WBC: white blood cell ( × 10^3^ cells μL^−1^); Hb: haemoglobin (g dL^−1^); HCT: hematocrit (%), MCV: mean cell volume (fl); MCH: mean cell haemoglobin (pg); MCHC: mean cell haemoglobin concentration (g dL^−1^).

### 3.4 Alkaline phosphatase activity

**Fig. 2:**
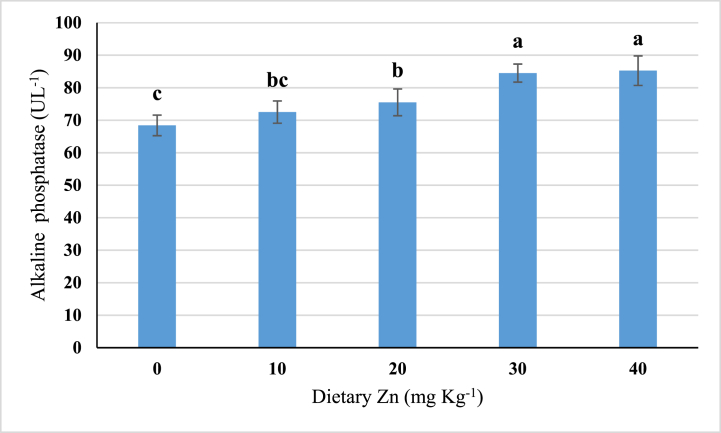
Serum alkaline phosphatase activity in stinging catfish fed with graded levels of Zn. Different superscript letters indicate significant differences (*P* < 0.05) among the treatments (means in triplicate ± SD).

The serum alkaline phosphatase (ALP) activity of stinging catfish after 10 weeks of feeding trial is presented in Fig. 2. In this study, the ALP level was found to increase with increasing dietary Zn supplementation up to 30 mg Zn kg^−1^ diet. Further increase in dietary Zn supplementation, i.e., 40 mg Zn kg^−1^ diet, did not show a similar trend of increase in serum ALP.

## 4 Discussion

### 4.1 Growth performance and feed conversion ratio in stinging catfish

It has been reported that Zn is a vital micronutrient for fish, being necessary for good somatic growth [31], with Zn deficiency causing decreased levels of IGF-I, growth hormone receptor, and mRNA protein binding growth hormone [[32], [33], [34]]. Therefore, this study was designed to find out the optimum dietary requirement of Zn for stinging catfish. The findings of this study revealed that the optimal Zn level in feed was necessary for the proper growth and physiological function of stinging catfish. Diets supplemented with Zn in the present study showed better growth performance (WG and SGR) and feed conversion ratio (FCR) in stinging catfish compared to the control diet. The highest WG, SGR, and the lowest FCR were recorded in fish fed with the diet supplemented with 30 mg kg^−1^ Zn. According to the second-order polynomial regression response, the optimum dietary inclusion level of Zn for maximum WG and SGR was calculated to be 27.4 and 28.6 mg Zn kg^−1^ diet, respectively. Similar dietary requirements have been reported in Nile tilapia, *Oreochromis niloticus,* fed with a diet supplemented with a graded level of Zn (from ZnSO_4_·7H_2_O) for 70 days [35].

In comparison of our findings, lower Zn requirement was recorded in other studies viz. 20 mg Zn kg^−1^ for channel catfish, *Ictalurus punctatus* [36], 20–25 mg Zn kg^−1^ for red drum, *Sciaenops ocellatus* [37], 17–21 mg Zn kg^−1^ for yellow catfish, *Pelteobagrus fulvidraco* [19]. However, a higher level of Zn requirement was also found in some experiments, i.e., 55 mg kg^−1^ for grass carp, *Ctenopharyngodon idella* [15], 37.1–52.4 mg kg^−1^ for adult Nile tilapia *O. niloticus* [38], 47.85–52.93 mg kg^−1^ for rui *Labeo rohita* [12]. The wide variability in Zn requirement values as above may be associated with the diﬀerences in ﬁsh species, life stages, forms of Zn supplementation, Zn concentrations of rearing water and experimental designs, and diﬀerent statistical models for estimating Zn requirements. Dietary Zn at optimum level enhanced the growth performance of stinging catfish in the present study, indicating that the Zn was an essential element for the fish species, in agreement with the reports in African catfish *Clarias gariepinus* [39], Nile tilapia *O. niloticus* [35,40], rui *L. rohita* [41], and grass carp, *C. idella* [15]. This is because zinc actively participates in the synthesis and metabolism of nucleic acids as well as the metabolism of proteins, lipids, and carbohydrates [16]. According to Ref. [44], dietary Zn enhances fish aldolase, peptidase, and phosphatase enzyme activity. As a result, enhanced growth and nutrient utilization are associated with increased digestive enzyme activity [18]. Consequently, as an essential microelement, Zn improved the metabolism, antioxidant capacity, and muscular growth and ultimately resulted in better growth performance at optimum dietary level [11].

### 4.2 Mineral composition of bone and muscle

The zinc levels in fish bone and muscle increased as dietary zinc levels increased, with fish fed diets containing 30 mg Zn/kg exhibiting the maximum zinc contents. Nevertheless, additional increases in dietary Zn did not result in further increases in the zinc contents of bone and muscle. The bone and muscle Zn status is conservative because once tissue Zn saturation is achieved, adequate Zn should be available for all other physiological processes [36,42]. Likewise, Huang et al. [38] found an increase in bone and muscle Zn content of Nile tilapia until the optimum dietary supplementation level of Zn (52.1 mg kg^−1^) and then stabilized. Similar to our research, the Zn content of bone increased until the dietary Zn demands were satisfied in channel catfish *I. punctatus* [43], Nile tilapia [23], and red drum *S. ocellatus* [37] which supports the findings of the present study. The quantity of dietary Zn required by stinging catfish was determined by these response characteristics, and it was calculated through a regression model that a dietary Zn level of 36.5 and 34.1 mg kg^−1^ was sufficient to maintain the typical levels of Zn in the bone and muscle tissue (Fig. 1).

Opposite to Zn concentration, Fe and Cu concentrations in bone and muscle was inversely related to dietary Zn levels (Table 3). In this study, both Fe and Cu content of bone and muscle tended to significantly decrease in stinging catfish when dietary Zn was increased; because, during absorption in the digestive tract, Zn competes with other bivalent minerals like Cu, Fe, calcium, and cadmium for the same binding sites [20]. Similar to our study, dietary Zn levels impacted the levels of the tissue Fe and Cu contents of rui *L. rohita* [12] and hybrid tilapia *O. niloticus* × *O. aureus* [44], liver Fe of channel catfish *I. punctatus* [45] and whole-body Fe of Nile tilapia *O. niloticus* [35]. Certain indications of Zn–Cu antagonism have been found in rainbow trout *Salmo gairdneri* by Ref. [46]. The decrease in Cu levels caused by the high affinity of Cu for the metal binding sites on metallothionein (MT) could replace Zn and take on a form less likely to be transported into the blood. It has been reported that Zn can antagonize Fe because Fe and Zn prevent each other from being absorbed by the digestive tract [47].

### 4.3 Haematological response

There was a significant difference in RBC, WBC, hemoglobin, and hematocrit count among the treatments in the present investigation. An increase in RBC, hemoglobin, and hematocrit percentage is a useful indicator of the good immunological sign of stinging catfish because it indicates improved oxygen transportation capacity of fish. Understanding fish nutrient dynamics and the effects of diet on fish health and physiology increasingly depends on hematological parameters since they can reveal vital information about these effects [[48], [49], [50]]. However, there is scarce information concerning the blood parameters of stinging catfish, especially under culture. Zn plays a crucial function in hemoglobin synthesis by activating d-aminolevulinic acid dehydrogenase, a critical enzyme needed for the development of porphobilinogen from two molecules of d-aminolevulinic acid [20]. Additionally, Zn is a necessary mineral that influences the synthesis of proteins in red blood cells, including superoxide dismutase and carbon dioxide, both of which are necessary for the smooth functioning of erythrocytes [20,38,51]. In the current investigation, increasing the Zn inclusion level to 30 mg kg^−1^ considerably boosted RBC counts before reaching a plateau. Dietary Zn supplements had impacts on RBC counts, hemoglobin, and hematocrit values, similar to our study, in Nile tilapia *O. niloticus* [52] and rui *L. rohita* [12]. Moazenzadeh et al. [20] also reported considerable improvement in RBC count, hemoglobin, and hematocrit, and the highest values were noted at 46.4 mg kg^−1^ dietary Zn in Siberian sturgeon *Acipenser baerii*.

In the current investigation, the highest WBC count was found in fish fed 30 mg Zn kg^−1^. Fish fed the control diet had the lowest WBC count, whereas fish fed with an increasing Zn supplementation exhibited an increasing trend in WBC count. This rise in WBC count could be due to reduced zinc-induced liver, kidney, and gill tissue damage [53,54]. It has been found that Zn increased WBC counts in Mozambique tilapia *O. mossambicus* [55] and hybrid catfish *Heteroclarias* sp [56]. Dissimilar to this study, Thangapandiyan and Monika [57] did not find any difference in the WBC count of rui *L. rohita* in different experimental groups supplemented with graded levels of Zinc Oxide Nanoparticles (ZnONP). In the present study, the MCV and MCH count decreased with increasing dietary supplementation levels of Zn. Similar findings regarding MCV and MCH were also observed in Nile tilapia *O. niloticus* [44], rui *L. rohita* [12], and in Mozambique tilapia *O. mossambicus* [55]. According to Hrubec and Smith [58], more active fish have greater hemoglobin levels and lower MCV. Therefore, the stinging catfish fed the higher dietary Zn levels had lower MCV, demonstrating their greater resistance to hypoxia, which frequently occurs in the culture state. However, in the present study, the MCHC of fish fed with supplementary Zn showed increasing trends with increasing dietary Zn levels. Similarly, Zn treatment led to a considerable increase in MCHC levels in Mozambique tilapia *O. mossambicus* [55]. MCHC is the indication of the average concentration of haemoglobin in RBC cells. An increase in Hb with the increase in dietary Zn supplementation might be the reason for the increased MCHC.

### 4.4 Serum alkaline phosphatase activity

The serum alkaline phosphatase (ALP) level of stinging catfish juveniles was improved with a higher dietary Zn supplementation level. Serum alkaline phosphatase activity is a reliable Zn status indicator [59]. In an experiment on juvenile grass carp *C. idella*, Liang et al. [15] discovered that fish fed a diet deficient in Zn had considerably lower plasma ALP activity. In another study, channel catfish *I. punctatus* [36] and Nile tilapia *O. niloticus* [38] responded similarly. According to Gatlin and Wilson [36], ALP significantly increased (*P* < 0.05) with an increasing level of dietary Zn from 0 to 60 mg Zn kg^−1^ diet in fingerling channel catfish. On the other hand, Li and Huang [44] reported that in hybrid tilapia, *Oreochromis niloticus* × *O. aureus* ALP increased with increasing dietary Zn levels while further addition of Zn decreased ALP activity. Therefore, these previous results support the ALP value of this study.

## 5 Conclusion

The study's findings established a strong base for supplying optimum levels of dietary Zn as an essential micronutrient in the diet of stinging catfish. The best growth performance, feed utilization, haematological profile, and bone and muscle mineralization of stinging catfish were observed in the diet supplemented with 30 mg Zn kg^−1^. However, according to regression analysis, optimum dietary supplementations of Zn for maximum WG and SGR, bone, and muscle Zn concentrations of stinging catfish were determined to be 27.4, 28.6, 36.5, and 34.1 mg Zn kg^−1^ diet, respectively. Therefore, the recommendable dietary inclusion level of Zn ranged between 27.4 and 36.5 mg kg^−1^ in the practical diet for stinging catfish.

## Funding

This research was funded by the University Grants Commission of Bangladesh.

## Ethical approval

The authors followed all applicable international, national, and institutional guidelines for the care and use of fish.

## Data availability

Data will be made available on request.

## CRediT authorship contribution statement

**Muslima Akter Lima:** Writing – original draft, Methodology, Investigation, Formal analysis, Conceptualization. **Md. Amzad Hossain:** Writing – review & editing, Supervision, Project administration, Funding acquisition, Conceptualization. **Md. Rabiul Islam:** Writing – review & editing. **Md. Nur Amin Mukul:** Writing – review & editing. **Taslima Akter:** Writing – review & editing, Supervision.

## Declaration of competing interest

The authors declare the following financial interests/personal relationships which may be considered as potential competing interests: Md. Amzad Hossain reports financial support was provided by University Grants Commission of Bangladesh. If there are other authors, they declare that they have no known competing financial interests or personal relationships that could have appeared to influence the work reported in this paper.
